# Recurrent myelitis after allogeneic stem cell transplantation. Report of two cases

**DOI:** 10.1186/1471-2377-10-76

**Published:** 2010-09-01

**Authors:** Martin Voß, Felix Bischof

**Affiliations:** 1 Hertie Institute for Clinical Brain Research, Department of General Neurology, University of Tübingen, Hoppe-Seyler-Strasse 3, 72076 Tübingen, Germany

## Abstract

**Background:**

Allogeneic and autologous haematopoietic stem cell transplantation are established treatment options for haematological malignancies and may possibly be employed to treat a range of genetic and autoimmune diseases.

**Case presentation:**

We report two patients who developed an acute myelitis within their thoracic spinal cord after allogeneic stem cell transplantation. Myelitis in these patients was not related to graft versus host disease or immune reconstitution and was responsive to intravenous methylprednisolone and cyclophosphamide.

**Conclusions:**

Myelitis is a possibly disabling consequence of haematopoietic stem cell transplantation.

## Background

Autologous and allogeneic haematopoietic stem cell transplantation (HSCT) are increasingly employed in the treatment of haematological malignancies and solid tumors. Allogeneic HSCT requires eradication of the patients bone marrow by high dose combination chemotherapy and total body irradiation and intravenous injection of stem cells isolated from the bone marrow or the blood of a related or unrelated human leukocyte antigen (HLA)- identical donor. After HSCT, immunosuppressive treatment is required to reduce the risk of transplant rejection and graft versus host disease (GvHD) [1].

Neurological complications after HSCT include metabolic encephalopathies, cerebral infarctions, bleedings and infections due to bone marrow depletion or immunosuppression. GvHD which develops in 40-70% of patients after allogeneic HSCT may manifest as polyneuropathy, myositis or myasthenia [1,2]. We report two patients who developed acute myelitis after allogeneic HSCT indicating that myelitis should be included in the list of possible neurological complications of HSCT.

## Case Presentation

### Case 1

A 57 year old male Caucasian was diagnosed with chronic myelo-monocytic leukaemia in April 2004. He was treated with hydroxycarbamide until April 2005 before an allogeneic unrelated peripheral stem cell transplantation was performed. During this procedure, he was treated with 40 mg/m^2 ^fludarabine and 130 mg/m^2 ^busulfan on day -6 to -3 [3]. GvHD prophylaxis was achieved with 20 mg/m^2 ^anti-thymocyte-globulin on day -3 to -1, 5 mg/m^2 ^methotrexate and tacrolimus (serum concentration 10 ng/ml). He did not show any signs of GvHD.

Four weeks after stem cell transplantation, he developed an acute numbness of both feet, weakness of his lower extremities and urinary difficulties and was admitted to our hospital. On admission, he had a mild paraparesis (MRC grade 4 in both legs) but was still able to walk. He had hypaesthesia and paraesthesia in both legs with a cranial level at L1 and difficulty urinating.

**Figure 1:**
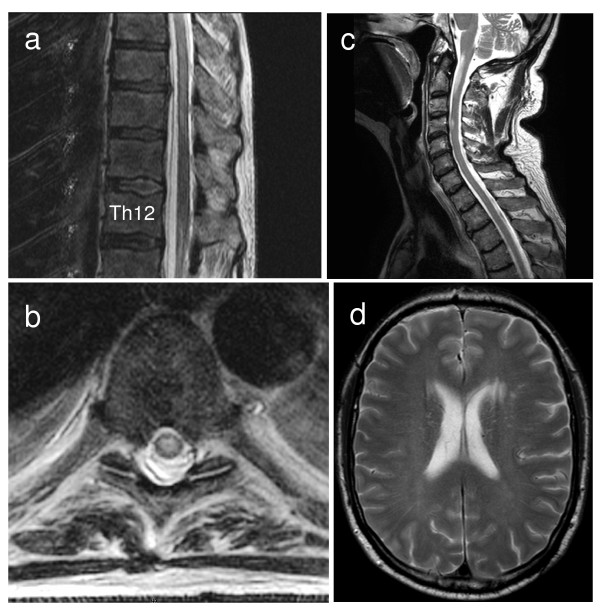
**MRI data of patient 1 (a, b) and patient 2 (c, d)**. Saggital (a) and axial (b) T2-weighted magnetic resonance images show increased signal intensity in the spinal cord of patient 1. The lesion extends from the T5 to the T10 vertebral body. The axial image shows the spinal cord at the level of the T6 vertebral body. (c) T2-hyperintense lesion on the level of the C4 vertebral body of patient 2. (d) Brain-MRI shows a single T2-hyperintense paraventricular left hemispheric lesion in patient 2.

Magnetic resonance imaging (MRI) of the spinal cord showed a hyperintense lesion on T2-weighted images extending from the T5 to the T10 thoracic vertebral body which was not contrast enhancing (Figure 1a, b). Cerebral MRI did not show any hyperintense lesions on T2- or FLAIR-weighted images and no contrast enhancement. Cerebrospinal fluid (CSF) analysis revealed an increased protein concentration, normal cell count and negative oligoclonal bands. PCR of CSF was negative for CMV, HSV-1, HSV-2, VZV, HHV-6 and HHV-7 DNA. Flow cytometric analysis of peripheral blood cells was normal and without evidence for recurrence of leukaemia. Based on these findings, a diagnosis of acute myelitis was made and the patient was treated with 500 mg methylprednisolone i.v. per day for five days. Within the following weeks, the symptoms resolved except for a hypoesthesia in both feet. Immunosuppressive treatment with Tacrolimus was switched to cyclosporin (serum concentration 200 ng/ml) and stopped after two weeks. After that, the neurological status of the patient remained constant for 1 ½ years. The patient was admitted again in February 2006 with a new weakness and numbness in his legs. On examination, he had a mild paraparesis (MRC grade 4), his knee and ankle reflexes were brisk and plantar responses were normal. He had hypaesthesia of his legs and trunk with a cranial level at Th12. He was able to stand independently and walk a few steps with walking aids. MRI showed a new T2 hyperintense lesion within the spinal cord extending from the T3 to the T5 thoracic vertebral body which was partially contrast enhancing. Symptoms improved after i.v. treatment with 1000 mg methylprednisolone per day for 5 days and tapering doses of oral prednisolone for 4 weeks. After that, he was able to walk 500 meters with walking aids. In November 2006 the patient complained again about a progressive weakness of the legs with a walking distance of 100 meters. On examination, he had a mild paraparesis (MRC grade 4 in both legs). Knee and ankle reflexes were exaggerated and Babinski's sign was positive on the left side. Sensitivity to light touch was absent and position sense was markedly diminished in both feet. MRI of his spinal cord showed a new hyperintense lesion on T2-weighted images in the cervical spinal cord at the level of the C3 vertebral body. The patient was treated with 750 mg/m^2 ^intravenous cyclophosphamide every four weeks for seven months. During the first five infusions, the weakness and numbness of his legs improved and subsequently remained stable. He was able to walk 500 meters at that time. Upon repeated follow-up visits over a period of three years, the neurological status was stable and control MRI scans of the spinal cord did not show any new lesions.

### Case 2

A 65 year old Caucasian developed acute myeloic leukaemia in October 2006. Two months later, allogeneic HSCT of a HLA-identical related donor was performed after conditioning with 30 mg/m^2 ^fludarabine, 100 mg/m^2 ^amsacrine and 2000 mg/m^2 ^ara c on days -12 to -9, 40 mg/kg cyclophosphamide on days -5 and -4, 10 mg/kg anti-thymocyte globulin on days -4 to -2 and 4 Gy total body irradiation. He received cyclosporin and mycophenolat mofetil for GVHD prophylaxis and remained in complete remission during repeated follow up visits. In September 2009, he noticed a weakness and hypoesthesia in his left foot and a few days later in his right foot and difficulties to urinate. Upon examination, he displayed a mild paraparesis (hip flexion and knee extension MRC grade 4) with normal reflexes and without pyramidal signs. Sensitivity to pain and temperature was diminished in both legs. MR tomography showed hyperintense lesions on T2 weighted images within the spinal cord at the level of the C2, C4 and T8 vertebral bodies which were partially contrast-enhancing (Figure 1c). CSF protein concentration was increased (55 mg/dl) and oligoclonal bands were positive. Anti-nuclear antibodies, antibodies against Borrelia burgdorferi and PCR for CMV, HSV-1, HSV-2 and VZV-DNA were all negative. Visually evoked potentials were normal. After high dose i.v. methylprednisolone therapy (1000 mg methylprednisolone per day for five consecutive days), the symptoms resolved completely. The weakness in both legs appeared again in October 2009. MRI showed a T2 hyperintense, contrast enhancing left-hemispheric lesion (Figure 1d) and contrast enhancement of the lesion within the cervical spinal cord. The symptoms resolved completely after high dose intravenous methylprednisolone therapy.

## Conclusions

According to recent publications, about 16% of patients with autologous and allogeneic HSCT develop neurological complications [1,2]. These complications include drug related encephalopathies, seizures, cerebral infarctions and haemorrhages that result from bone marrow depletion and infections during immunosuppression. Within months or years after HSCT, CNS manifestations of the original disease and secondary neoplasms may develop.

The diagnosis of acute myelitis was based on T2 hyperintense signals in spinal cord MRI, increased CSF protein concentration and oligoclonal bands. The fact that no infectious agent could be identified, the responsiveness to i.v. methylprednisolone and the fact that the condition recurred at different levels of the spinal cord further support this diagnosis and rule out other diagnoses including glioma, infarctions or viral infections of the spinal cord. Myelitis in these patients could possibly be a manifestation of CNS vasculitis, a heterogeneous group of diseases that include primary CNS vasculitis and vasculitis associated with connective tissue disease like systemic lupus erythematodes and Behcet disease. However, primary CNS vasculitis only rarely involves the spinal cord and is characterized by cerebral infarcts and irregularly configured cerebral vessels on brain MRA. In addition, the patients did not show any systemic symptoms of connective tissue disease. Myelitis as a manifestation of CNS vasculitis is thus unlikely.

Patient 2 showed a single T2 hyperintense lesion on cerebral MRI and in combination with the three lesions within the spinal cord would fulfill the diagnostic criteria for relapsing multiple sclerosis. However, the age at disease onset and the apparently self limiting course argue against this diagnosis.

One frequent complication of HSCT is acute or chronic GvHD which most commonly affects the skin, the intestinal mucosa and the liver. Both patients did not show any clinical signs of GvHD indicating that myelitis in these patients is not a manifestation of GvHD. In addition, myelitis after HSCT could be a result of immune reconstitution similar to the immune reconstitution inflammatory syndrome (IRIS) that has been well characterized in HIV infected individuals under highly active anti-retroviral therapy but has also been reported to occur after HSCT [4]. However, the fact that myelitis in patient one developed years after reestablishment of the immune system and the fact that myelitis had a remitting course in both patients argues against this possibility.

Alternatively, myelitis in these patients could be caused by an immune attack of the donors HLA-identical lymphocytes against the hosts CNS-tissue and this could indicate an inherent risk of allogeneic HSCT to induce autoimmune phenomena. It would be important to know whether this is indeed the case, because HSCT is considered to be a treatment option for autoimmune diseases including severe forms of multiple sclerosis [5,6]. Multiple sclerosis is believed to be mediated by self-directed lymphocytes and allogeneic HSCT offers the advantage of replacing the endogenous autoreactive immune cell repertoire with new, possibly less autoreactive immune cells. The presented cases now indicate that allogeneic HSCT carries the risk to induce autoimmune CNS-disease with potentially disabling consequences. The incidence of myelitis after HSCT may be higher than previously estimated because similar cases may not have been reported and the incidence may vary between different ethnic groups.

These two cases demonstrate myelitis as a possible complication of HSCT. Myelitis was not a result of GvHD or immune reconstitution and was successfully treated with high dose intravenous methylprednisolone and intravenous cyclophosphamide.

## Abbreviations

HSCT: haematopoietic stem cell transplantation; GvHD: graft versus host disease.

## Competing interests

The authors declare that they have no competing interests.

## Authors' contributions

MV and FB wrote the manuscript and approved its final form.

## Consent

Both patients provided written consent to publish this report.

## Pre-publication history

The pre-publication history for this paper can be accessed here:

http://www.biomedcentral.com/1471-2377/10/76/prepub
